# Arginase Treatment Prevents the Recovery of Canine Lymphoma and Osteosarcoma Cells Resistant to the Toxic Effects of Prolonged Arginine Deprivation

**DOI:** 10.1371/journal.pone.0054464

**Published:** 2013-01-24

**Authors:** James W. Wells, Christopher H. Evans, Milcah C. Scott, Barbara C. Rütgen, Timothy D. O'Brien, Jaime F. Modiano, Goran Cvetkovic, Slobodan Tepic

**Affiliations:** 1 Center for Advanced Orthopaedic Studies, Beth Israel Deaconess Medical Center, Harvard Medical School, Boston, Massachusetts, United States of America; 2 Masonic Cancer Center, University of Minnesota, Minneapolis, Minnesota, United States of America; 3 Central Laboratory, Department of Pathobiology, University of Veterinary Medicine Vienna, Vienna, Austria; 4 Veterinary Population Medicine Department and Stem Cell Institute, University of Minnesota, Saint Paul, Minnesota, United States of America; 5 Kyon AG, Zurich, Switzerland; University of North Carolina at Chapel Hill, United States of America

## Abstract

Rapidly growing tumor cells require a nutrient-rich environment in order to thrive, therefore, restricting access to certain key amino acids, such as arginine, often results in the death of malignant cells, which frequently display defective cell cycle check-point control. Healthy cells, by contrast, become quiescent and remain viable under arginine restriction, displaying full recovery upon return to arginine-rich conditions. The use of arginase therapy to restrict available arginine for selectively targeting malignant cells is currently under investigation in human clinical trials. However, the suitability of this approach for veterinary uses is unexplored. As a prelude to in vivo studies in canine malignancies, we examined the in vitro effects of arginine-deprivation on canine lymphoid and osteosarcoma cell lines. Two lymphoid and 2 osteosarcoma cell lines were unable to recover following 6 days of arginine deprivation, but all remaining cell lines displayed full recovery upon return to arginine-rich culture conditions. These remaining cell lines all proved susceptible to cell death following the addition of arginase to the cultures. The lymphoid lines were particularly sensitive to arginase, becoming unrecoverable after just 3 days of treatment. Two of the osteosarcoma lines were also susceptible over this time-frame; however the other 3 lines required 6–8 days of arginase treatment to prevent recovery. In contrast, adult progenitor cells from the bone marrow of a healthy dog were able to recover fully following 9 days of culture in arginase. Over 3 days in culture, arginase was more effective than asparaginase in inducing the death of lymphoid lines. These results strongly suggest that short-term arginase treatment warrants further investigation as a therapy for lymphoid malignancies and osteosarcomas in dogs.

## Introduction

Arginine is an amino acid that plays a critical role in the urea cycle, the process by which the body prevents the build-up of toxic ammonia. Arginine also plays a key role in several other complex metabolic pathways. In addition to being a precursor of nitric oxide, urea, ornithine and agmatine, arginine is also necessary for the synthesis of creatine, polyamines, citrulline, and glutamate.

Twenty-five years ago, Tepic suggested the use of controlled essential amino acid deprivation as a means of selective attack on tumor cell populations (personal communications to: Mikic, 1987; Wheatley, Perren, Mikic, Mann, 1989). Tumor cells have a high requirement for arginine [1] and subsequently arginine-restriction has been studied for many years as a means to control the growth of, or induce the death of, malignant tumor cells. Healthy cells deprived of arginine are believed to arrest their cell cycle in G_1_ [2], and enter into a quiescent state from which they can fully recover following the reintroduction of arginine. In contrast, malignant cells often lack stringent cell cycle checkpoint control and continue to cycle past G_1_, which leads to cell death [3]. Arginine-deprivation is therefore of interest as a means to selectively target tumor growth and survival, and the effects of arginine-deprivation have subsequently been extensively studied using both mouse and human cell lines [4]. Phase 1/2 clinical trials are presently underway in patients with hepatocellular carcinoma in which arginine levels are depleted using a PEGylated form of arginase [5] or arginine deiminase [6].

Arginine is considered to be a semi-essential amino acid in humans since the body cannot make enough of it during periods of fast growth, requiring that it be supplied from external sources like other essential amino acids [1]. However in felines, who are obligate carnivores, arginine is an essential amino acid and its removal from a single meal can cause emesis, tetanic spasms, and death [7]. Although also in the order *Carnivora*, the dog has evolved to eat an omnivorous diet [8] and is known to be capable of synthesizing arginine, raising the possibility that, unlike in the cat, arginine manipulation may be tolerated to some extent in the dog, a species that is particularly prone to lymphoma and osteosarcoma.

Lymphoma is one of the three most common cancers of dogs, accounting for 20–25% of all canine tumors with an average lifetime risk of ∼1 in 15 [9]. The disease affects dogs of virtually every age and breed without a significant gender bias. However, there are breed predispositions that suggest heritable risk factors contribute to lymphoma risk. boxers, golden retrievers, Labrador retrievers, cocker spaniels and bassett hounds are among the breeds that have an increased risk for lymphoma, while others such as Chihuahuas, dachshunds and Pomeranians show a lower incidence of the disease [10], [11], [12]. The contribution of heritable factors is further supported by the familial clustering observed in certain Rottweiler and Scottish terrier lines [13], by the fact that breed might influence response to therapy [14], and by the observed phenotype bias among certain breeds [15].

Osteosarcoma is another prevalent tumor that shows remarkable breed predilection in dogs [16]. It occurs mostly in large and giant breeds, with lifetime risk in some breeds estimated to be as high as 20% with the total number of cases diagnosed per year in the US alone exceeding 8,000 [17], [18], [19]. The effects of arginine restriction on the growth and survival of canine lymphoma and osteosarcoma cells remains unknown.

In this study, we investigated the sensitivity of canine lymphoid and osteosarcoma cells to cell death induced by arginine restriction in vitro as a prelude to possible clinical trials in dogs. We show that although a prolonged period of arginine restriction uniformly halted the growth of these transformed cell lines, the majority of the lines did not die and were capable of mounting a full recovery upon the reintroduction of arginine. While a minority of lines were capable of utilizing citrulline to continue their growth during arginine deprivation, no cells were capable of utilizing ornithine in this manner. The addition of arginase prevented the recovery of all cell lines tested, suggesting that trace levels of arginine associated with these cells allows them to survive periods of culture in arginine-free medium. Canine lymphomas appeared to be particularly susceptible to arginase, and in contrast to asparaginase - an enzyme commonly used in the treatment of human leukemia and canine lymphoma - no canine lymphomas were able to recover following just 3 days of arginase treatment.

## Materials and Methods

### Cell lines

The canine lymphoid cell lines: GL-1 (B-cell leukemia) [20], CLBL-1 (B-cell lymphoma) [21], 17–71 (B-cell lymphoma) [22], CL-1 (T-cell leukemia) [23] and OSW (T-cell leukemia) [24], the murine lymphocytic leukemia cell line L1210 (purchased from the ATCC), and the canine osteosarcoma cell lines (all supplied by J.F.M): D-17, OSCA-2, OSCA-8, OSCA-29, OSCA-40, OSCA-71, and OSCA-78 [25] were maintained in RPMI 1640 culture medium (Mediatech Inc., Herndon, VA, USA) supplemented with 10% fetal bovine serum (FBS, Thermo Scientific, South Logan, UT, USA), 2.05 mM L-glutamine (Mediatech Inc), 100 U/ml penicillin, 100 µg/ml streptomycin, and 250 ng/ml amphotericin (Antibiotic-Antimycotic; Life technologies, Carlsbad, CA, USA) at 37°C/5% CO_2_.

### Multipotent Adult Progenitor Cells (MAPC) isolation and culture

Bone marrow (BM) was harvested from the femur of a healthy, adult (1–3 years old), large-breed mongrel dog using a percutaneous Jamshidi bone marrow aspiration needle under general anesthesia. The dog was housed at the Research Animal Resources Facility, Minneapolis Campus, University of Minnesota, and the BM harvest was carried out as per the guidelines of the University of Minnesota Committee on the Use of Animal Subjects in Research. BM mononuclear cells were obtained by Ficoll-Paque density gradient centrifugation (Sigma Chemical, St. Louis, MO, USA).

Mononuclear cells were resuspended in 15 ml expansion medium consisting of 59% low-glucose DMEM (Gibco-BRL), 39% MCDB-201 (Sigma), 2% FCS, 1× insulin transferring selenium, 1× linoleic acid bovine serum albumin, 10 nM dexamethasone (Sigma), 100 U/ml penicillin and 1000 U/ml streptomycin (Gibco), and plated in T150 flasks coated with 10 ng/ml fibronectin. The expansion media was supplemented with 10 ng/ml Epidermal Growth Factor (Sigma) and 10 ng/ml platelet-derived growth factor BB (R&D Systems, Minneapolis, MN, USA). Cells were initially plated at a density of 500 cells/cm^2^ and allowed to grow to a density of approximately 1,500–2,500 cells/cm^2^ before being detached with 0.25% trypsin-EDTA (Mediatech) and replated at the initial seeding density.

### Assay Reagents

Arginine-free RPMI was specially formulated by the UCSF Cell Culture Facility, University of California, San Francisco, CA, and confirmed to be arginine-free using the Amino-Acid Analysis protocol below (Fig. S1A). Assay media comprised of RPMI (arginine-free or commercially available RPMI as indicated) supplemented with 1× antibiotic-antimycotic (Life technologies) and either 10% dialyzed fetal bovine serum (added to arginine-free media; dialyzed by ultrafiltration against 0.15 M NaCl with a 10,000 MW cut-off, Sigma) or 10% fetal bovine serum (“normal serum”; added to commercially available RPMI; HyClone Laboratories Inc., Logan, UT, USA). L-arginine, L-citrulline, L-ornithine monohydrochloride, L-asparagine, Asparaginase (from Escherichia coli) and L-arginase (from bovine liver) were also purchased from Sigma. Amino acid and enzyme stock solutions were made up immediately before use in warm assay media as indicated and filter sterilized using a 0.2 µm syringe filter.

### Arginine-Deprivation Assay

Non-adherent lymphoid cell lines, adherent MAPC or osteosarcoma cells (recovered using 0.05% trypsin, 0.5 mM EDTA buffer (Life technologies)) were harvested and washed twice with arginine-free RPMI, and then plated in multiple 96-well opaque plates (one for each time point, BD Falcon) at 10,000 cells/well (except: CL-1 cells: 20,000 cells/well; CLBL-1 cells: 100,000 cells/well; and MAPC: 5,000 cells/well) in 100 µl of assay media. A further 100 µl of assay media containing twice the final indicated concentration of arginine, citrulline, ornithine, asparaginine, arginase, or asparaginase was then added to each well. All samples were set up in triplicate and plates were incubated at 37°C/5% CO_2_. The metabolic activity of each well was assessed using the Luminescent Cell Viability Assay (below). Preliminary experiments measuring arginase activity over time showed that >78% of enzyme activity was lost after 3 days of culture at 37°C (data not shown).

In order to return the cells to arginine-containing culture conditions, wells containing arginase or asparaginase were incubated for 30 minutes at 37°C with 10 mM arginine or 10 mM asparaginine, respectively, to quench enzyme activity. All wells were subsequently washed twice with fresh assay media containing 1 mM arginine (plates were spun at 300 g for 5 minutes between washes), and returned to 37°C/5% CO_2_.

### Enzyme activity

Arginase and asparaginase activities were analyzed over time at 37°C/5% CO_2_ in the absence of cells to determine whether these enzymes maintained activity over the course of each experiment. At the relevant time point, 100 µl of 10 U/ml enzyme was removed from the incubator and mixed with 100 µl of assay media containing 10 mM arginine or asparaginine. The mixture was incubated at 37°C for 30 minutes prior to freezing at −80°C for analysis of amino acid content (see below).

### Cell-associated arginase activity

GL-1 cells (1×10^6^ cells/ml/well) were cultured overnight at 37°C/5% CO_2_ in assay media plus/minus 10 U/ml arginase. The cells were then washed twice in ice-cold PBS and spun at 1000 g/4°C for 10 minutes. Cell pellets were lysed for 10 minutes in 100 µl of 10 mM Tris-HCL containing 1 µM pepstatin A, 1 µM leupeptin, and 0.4% Triton X-100 (all from Sigma). The lysate was then passed through a 4 mm-diameter 0.2 µm cellulose acetate syringe filter (Thermo Scientific) and frozen at −80°C. Arginase activity in cell lysates and culture supernatants was assayed using the commercially available QuantiChrom Arginase Assay kit (BioAssay Systems, Hayward, USA) according to the manufacturer's instructions.

### Luminescent Cell Viability Assay

The presence of viable cells in culture was determined by measuring ATP, an indicator of metabolically active cells, using a commercially available kit (CellTiter-Glo Luminescent Cell Viability Assay, Promega U.S., Madison, WI, USA). Briefly, at the indicated time point culture plates were spun at 300 g for 3 minutes and 100 µl/well of supernatant was carefully removed. Plates were then incubated at room temperature for 30 minutes. 100 µl/well of the substrate solution was then added, and plates were mixed for 2 minutes on an orbital shaker to induce cell lysis. Following a further 10-minute incubation at room temperature the luminescence was recorded on Synergy MX Microplate Reader (Biotek, Winooski, USA).

For comparison to the Luminescent Cell Viability Assay, the enumeration of viable cells was also carried out using Trypan-Blue exclusion. Briefly, L1210 cells were seeded in triplicate wells of a 12-well plate at 1×10^5^ cells/well in 2 ml of test medium and incubated at 37°C/5% CO_2_. At the indicated time point the cells were resuspended and a 10 µl sample was mixed 1∶1 with Trypan-Blue. Viable cell counts were then determined using a hemacytometer.

### Amino Acid Analysis

Analysis of the amino acid content of cell culture supernatants was performed by the Functional Genomics Center Zürich. Samples were harvested at the indicated time points, filtered through a 0.2 µm filter, and frozen at −80°C before analysis. 20 µl/sample was deproteinized with 10% sulfosalicylic acid solution/250 µM norvaline, centrifuged for 5 min at 10,000 rpm, and derivatized using the MassTrak protocol (Waters Corp., Milford, USA). UPLC was performed, with standards every ten samples, using the MassTrak AAA chromatographic method (Waters Corp.). 1 µl per 100 µl product was injected on a MassTrak AAA 2.1×150 mm column on the ACQUITY UPLC System (Waters Corp.) according to the manufacturer's instructions.

### Statistical Analysis

All experiments were performed in triplicate unless otherwise indicated. Data are expressed as means ± standard deviation (SD). Comparisons of mean values were performed using unpaired Student's t-tests. P<0.05 (*) was considered significant. P<0.005 (**) and P<0.001 (***) are indicated.

## Results

### Cell viability correlates closely with metabolic activity

Arginine deprivation is known to cause the reversible cessation of cell growth by non-malignant cells, but is thought to lead to tumor cell death. During our studies we noted that canine lines deprived of arginine ceased dividing and did not resume cell division after arginine restoration. This suggested they had lost viability. However, they remained bright under phase contrast light microscopy and excluded trypan blue. Therefore, the assessment of cell viability by trypan blue exclusion was difficult.

**Figure 1:**
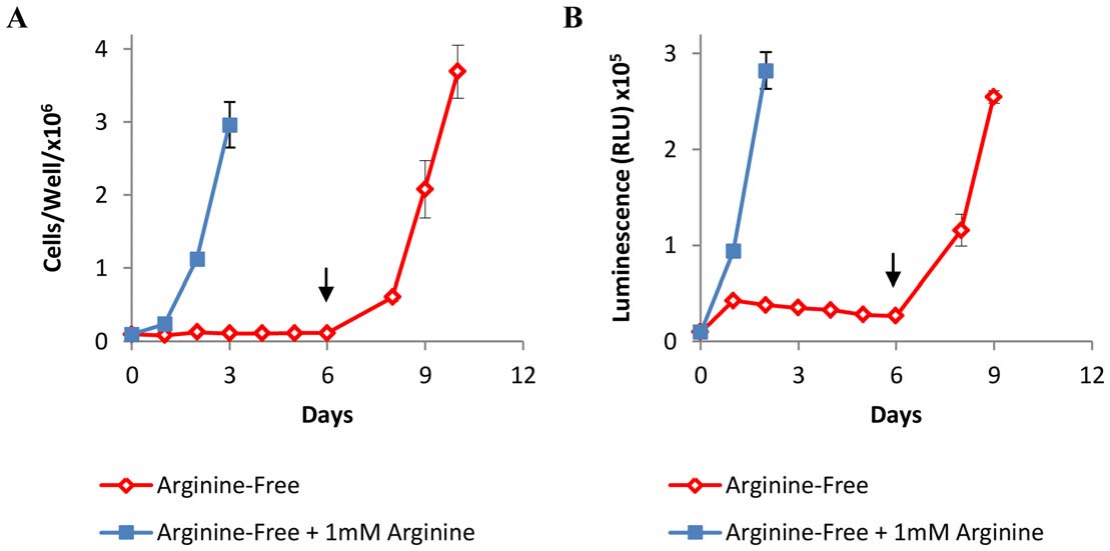
Cell viability within arginine-depleted cell populations. L1210 cells were cultured in arginine-free media with or without arginine supplementation for 6 days. Arginine was reintroduced into arginine-free cultures on day 6 as indicated by the arrows. Cell viability was determined by (A) Trypan blue exclusion, or (B) ATP quantification. For clarity, only data leading up to - and including – the maximal response is shown. Graphs show a representative experiment from three independent experiments with similar results. Error bars represent SD.

In order to accurately assess the effects of arginine withdrawal on cell viability we compared trypan blue exclusion (Fig. 1A) with the quantitation of ATP (Fig. 1B); an indicator of the energy charge of cells, using a highly sensitive luminescent cell viability assay. Murine L1210 cells were cultured in arginine-free medium with or without supplemental arginine (1 mM) for 6 days. Cells grown in the presence of supplemental arginine grew rapidly, whilst cells cultured in the absence of free arginine did not. Rather, these cells appeared quiescent, but continued to grow when arginine was returned to the culture on day 6. ATP quantitation showed a good correlation with trypan blue exclusion when assessing cell viability, and provided additional insight, not revealed by trypan blue exclusion, in detecting the gradual reduction of ATP between days 1 and 6 (Fig. 1B). Importantly, ATP quantification could be performed on adherent cells without the need for trypsinization. These data indicate that measurements of ATP concentration can be used as an alternative to trypan blue exclusion when assessing cell viability in the present context.

### The majority of canine lymphoid cell-lines and osteosarcomas can recover from prolonged arginine deprivation

**Figure 2:**
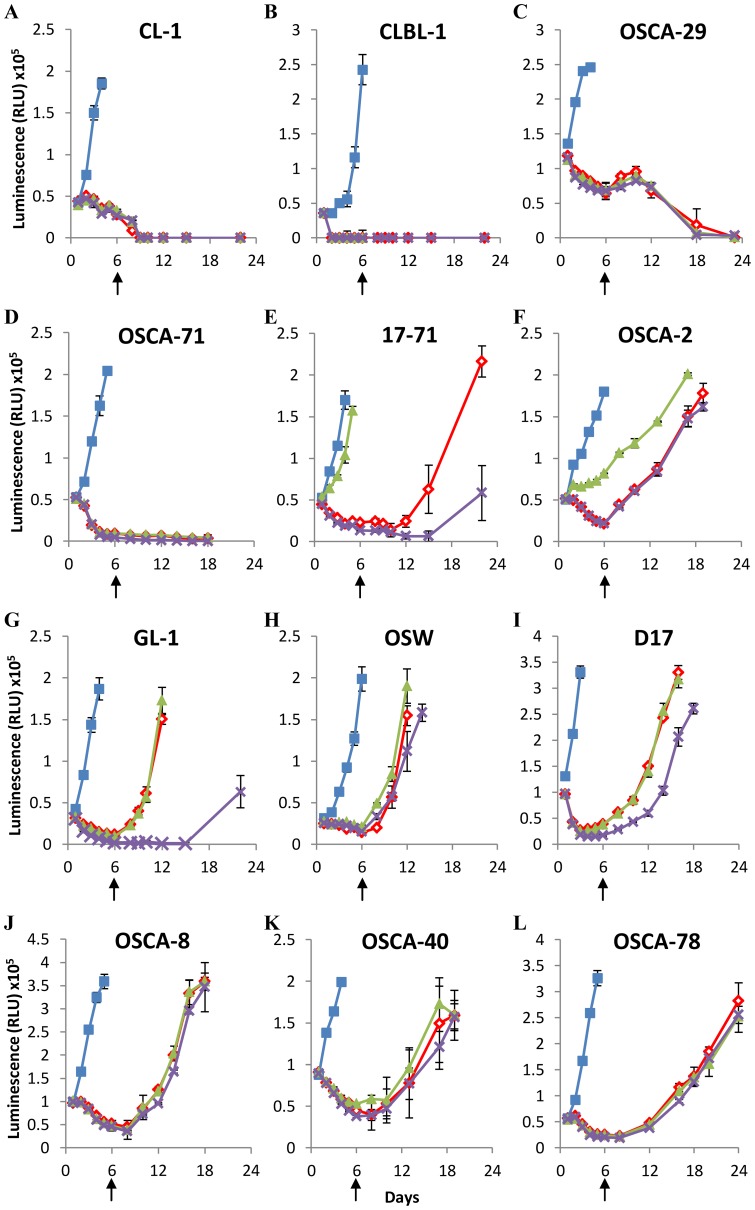
Sensitivity of canine lymphoid cells and osteosarcomas to prolonged arginine-deprivation. Canine lymphoid cells and osteosarcomas (A–L, described in Table 1) were cultured in Arginine-free media for 6 days with the amino acids indicated below. Arginine was reintroduced into cultures on day 6 as indicated by the arrows. Cell viability was determined by ATP quantification. Blue squares = +1 mM Arginine, Red diamonds = Arginine-free, Green triangles = +1 mM Citruline, Purple crosses = +1 mM Ornithine. Graphs show a representative experiment from three independent experiments with similar results. Error bars represent SD.

We assessed the recovery of 5 canine lymphoid cell lines and 7 canine osteosarcoma cell lines (described in Table 1) following 6 days of arginine-deprivation (Fig. 2A–L). We supplemented some arginine-depleted cultures with ornithine or citrulline from day 0 to assess the capacity of the cells to synthesize arginine from these amino acids, which requires the activity of ornithine transcarbamoylase to convert ornithine to citrulline, argininosuccinate synthetase to convert citrulline to argininosuccinate, and argininosuccinase to convert argininosuccinate to arginine. Amino-acid analysis of arginine-free media stocks confirmed the absence of arginine unless otherwise added as a supplement (Fig. S1A).

**Table 1:** Canine cell line characteristics.

Cell Line	Breed	Site	Tumor Type
17–71	*Unknown*	-	B-cell Lymphoma
GL-1	German Shepherd	-	B-cell Leukemia
CL-1	Japanese Terrier	-	T-cell Leukemia
OSW	Airedale Terrier	-	T-cell Leukemia
CLBL-1	Bernese Mountain Dog	-	B-cell Lymphoma
D17	Standard Poodle	Lung	Osteogenic sarcoma
OSCA-2	Rottweiler	*Unknown*	Osteosarcoma
OSCA-8	Rottweiler	Left Shoulder	Osteosarcoma
OSCA-29	Rottweiler	Right Humerus	Osteosarcoma
OSCA-40	Saint Bernard	Right Distal Femur	Osteosarcoma
OSCA-71	Golden Retriever	*Unknown*	Osteosarcoma
OSCA-78	German Shepherd	Right Distal Femur	Osteosarcoma

Two lymphoid cell lines (CL-1 and CLBL-1) and 2 osteosarcoma cell lines (OSCA-29 and OSCA-71) were found to be acutely sensitive to arginine depletion, and could not be recovered following 6 days of arginine deprivation, although each of these lines could be recovered following 3 days of arginine deprivation, indicating that the length of arginine deprivation is a critical factor for these lines (Fig. S2). However, the remaining cell lines all showed full recovery when arginine was added back to the culture following 6 days of arginine deprivation (indicated by arrows in Figure 2). One lymphoid cell line (17–71; Fig. 2E) and one osteosarcoma cell line (OSCA-2; Fig. 2F) were able to utilize citrulline to maintain cell growth in the absence of arginine. However, no cells were able to utilize ornithine in this manner, which corroborates previous observations examining non-hepatic cells [26]. The data show that canine lymphoid and osteosarcoma cell lines display different sensitivities to arginine deprivation in culture, and indicate that many canine cell lines subjected to prolonged culture in the absence of supplemental arginine can fully recover when arginine is made available.

### Arginase supplementation prevents the recovery of cell lines cultured under arginine-free conditions

**Figure 3:**
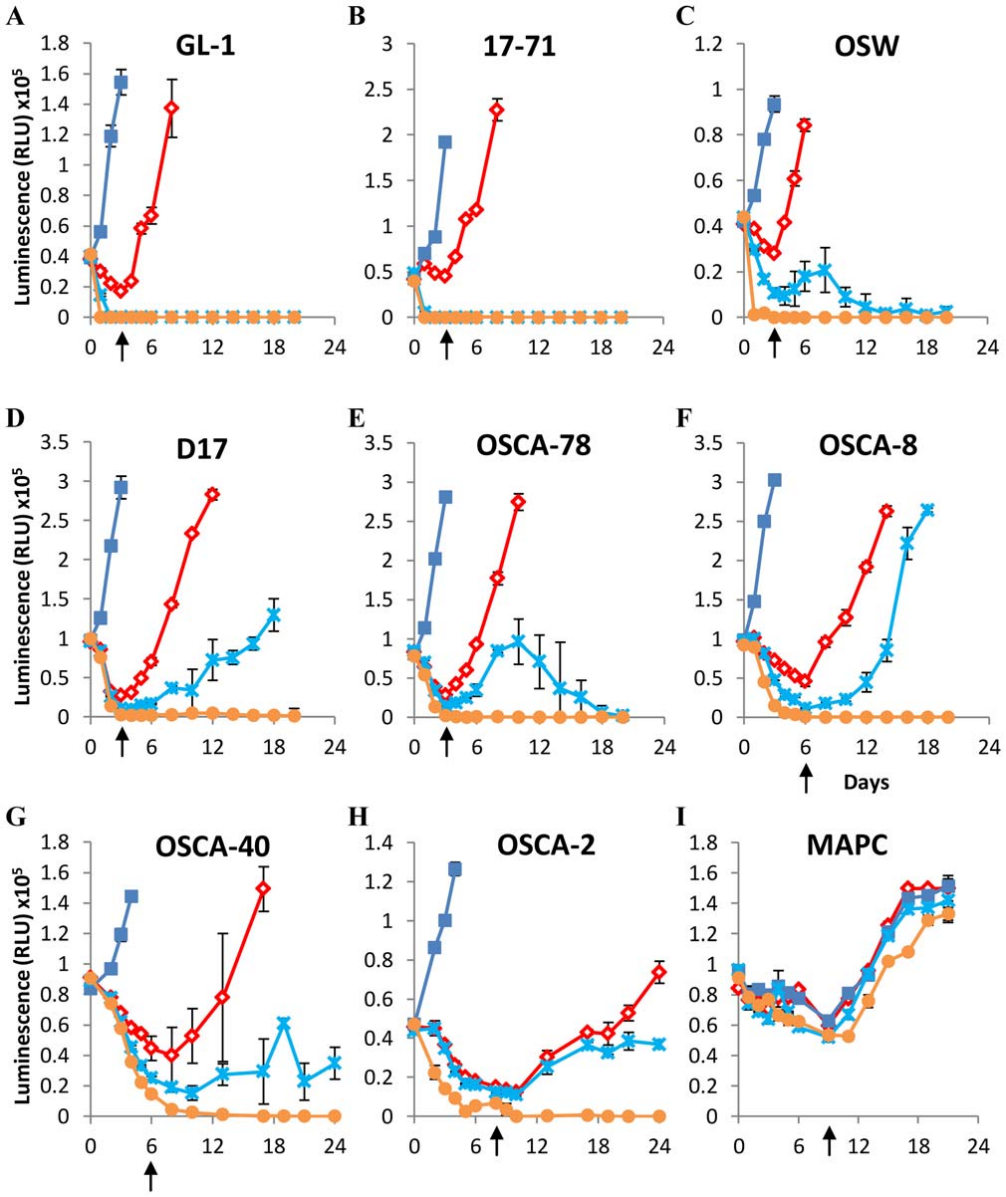
Arginase supplementation can prevent recovery of canine lymphoid cells and osteosarcomas subjected to arginine-deprivation. Canine lymphoid cells and osteosarcomas (A–H), and MAPC (I) were cultured in Arginine-free media for 3 days with or without arginase supplementation as indicated below. Arginase was removed and arginine reintroduced into cultures on day 3 (A–E), day 6 (F, G), day 8 (H), or day 9 (I) as indicated by the arrows. Cell viability was determined by ATP quantification. Blue squares = +1 mM Arginine, Red diamonds = Arginine-free, Blue crosses = +1 U/ml Arginase, Orange circles = +10 U/ml Arginase. Graphs show a representative experiment from three independent experiments with similar results. Error bars represent SD.

Although our cell cultures utilized specially formulated arginine-free media supplemented with dialyzed fetal calf serum, the possibility remained that trace levels of arginine present in the cultures contributed to the survival of some cell lines. We therefore investigated the effects of additional culture supplementation with arginase (Fig. 3A–H). At the time-points indicated by arrows in Figure 3, enzyme activity was quenched and the cells returned to arginine-replete culture conditions. The lymphoid cell lines GL-1, 17–71, and OSW (Fig. 3A–C) were acutely sensitive to arginase supplementation, and could not recover following just 3 days of culture with this enzyme. Recovery of the osteosarcoma cell-lines D17 and OSCA-78 (Fig. 3D–E) could also be prevented following 3 days of culture with the higher dose (10 U/ml) of arginase, but appeared to be more resistant to the effects of the lower dose of arginase (1 U/ml), although OSCA-78 cells did not appear to recover fully. OSCA-8 and OSCA-40 (Fig. 3F–G) required 6 days, and OSCA-2 required 8 days (Fig. 3H) of higher dose arginase culture to prevent recovery; however all three of these cell lines were relatively resistant to lower dose arginase culture over the same time period. In contrast, healthy control cells (Multipotent Adult Progenitor Cells; MAPC; Fig. 3I) derived from the bone marrow of a healthy dog recovered fully following 9 days of higher dose arginase culture, although these cells did not grow well for the initial 9-day period during which they were subjected to culture in dialyzed serum. Together, these data indicate that both lymphoid and osteosarcoma cell recovery can be prevented by arginase supplementation under conditions of arginine-deprivation, although osteosarcoma cells may vary widely in their susceptibility to this regimen.

### Arginase activity is cell-associated

**Figure 4:**
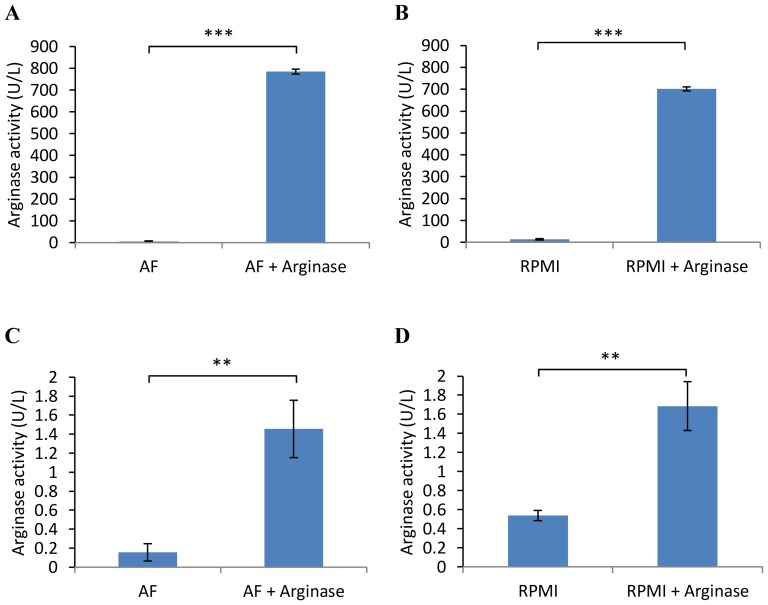
Arginase activity is cell-associated. GL-1 cells were cultured for 24 hours in arginine-free (AF) or normal RPMI media plus/minus 10 U/ml Arginase. Arginase activity was then measured in filtered supernatant (A, B) or cell lysate (C, D) using a QuantiChrom Arginase Assay kit. Graphs show a representative experiment from three independent experiments with similar results. Error bars represent SD.

Arginine was undetectable in the supernatant regardless of the presence or absence of arginase (Fig. S1B) raising the possibility that arginase killed cells by hydrolyzing intracellular arginine. Analysis of the amino acid content of cell lysate using the MassTrak AAA chromatographic method (used to similarly assess culture media) was not sensitive enough to address this question directly (data not shown). Therefore we assessed the level of arginase activity in cell lysates using a commercially available kit. To preclude the possibility of media-related artifacts, we compared cells grown in arginine-free media supplemented with dialyzed serum with cells grown in normal (commercially available) RPMI supplemented with normal (un-dialyzed) serum. As shown in Figure 4A–B, high levels of arginase activity could be measured in the supernatant of cultures supplemented with arginase, as expected. We also detected significantly increased levels of arginase activity in cell lysates of cells cultured in the presence of arginase (Fig. 4C–D). These data suggest that the effect of arginase in our cultures was not simply to convert free arginine in the media to ornithine. However, despite filtering out cell membranes it was not possible to conclusively determine whether the cell-associated arginase was actually being internalized by the cells or whether it remained on the cell surface.

### Arginase is more effective than asparaginase in short-term culture

**Figure 5:**
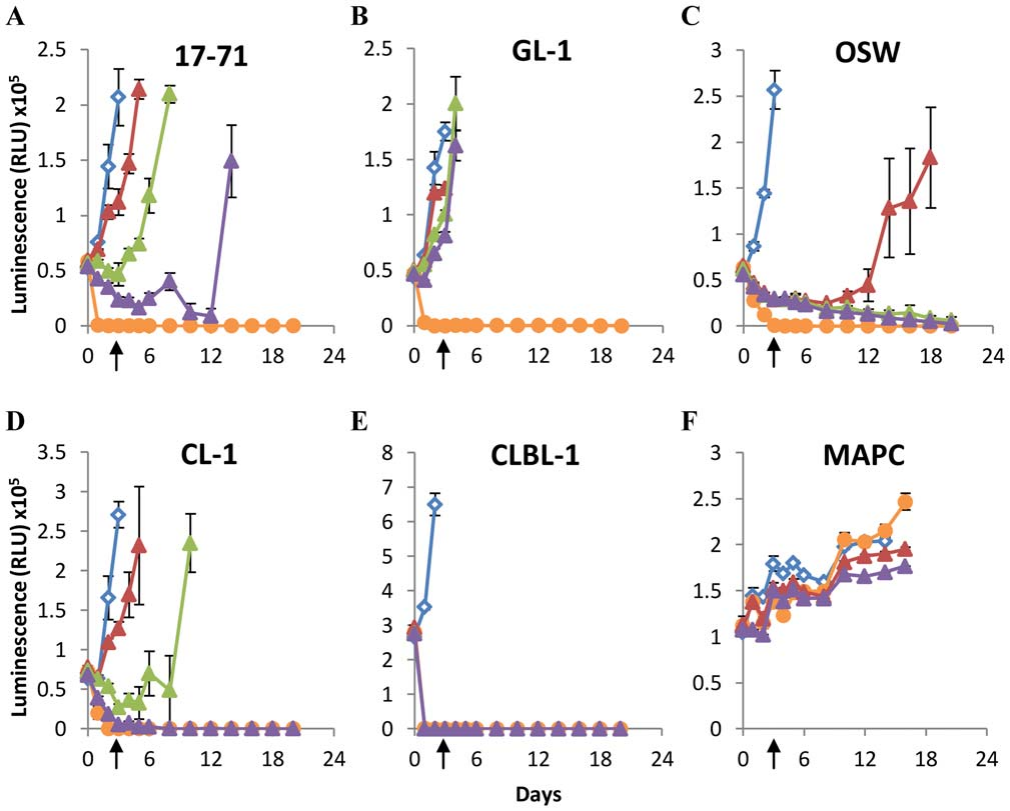
Comparison of Arginase and Asparaginase supplementation on the survival of canine lymphoid cells. Canine lymphoid cells (A–E), or MAPC (F) were cultured in normal RPMI for 3 days with or without arginase or asparaginase supplementation as indicated below. Enzymes were quenched on day 3 as indicated by the arrows. Cell viability was determined by ATP quantification. Blue diamonds = normal RPMI, red triangles = +0.3 U/ml Asparaginase, green triangles = +1 U/ml Asparaginase, purple triangles = +10 U/ml Asparaginase, Orange circles = +10 U/ml Arginase. Graphs show a representative experiment from three independent experiments with similar results. Error bars represent SD.

Asparaginase is an enzyme that has been commonly used in treatment protocols for leukemia for over 30 years. Since the canine lymphoid cell lines tested in our study appeared to be highly sensitive to arginase treatment, we compared the effectiveness of asparaginase and arginase over 3 days in culture. Cells were grown in standard RPMI containing 10% normal serum supplemented with the indicated dose of asparaginase or arginase (Fig. 5A–E). Of the 5 lymphoid lines tested, none were able to recover following 10 U/ml arginase treatment. In contrast, 2 out of 5 lines (17–71 and GL-1; Fig. 5A–B) appeared resistant to the highest dose of asparaginase (10 U/ml), and a further 2 lines (OSW and CL-1; Fig. 5C–D) appeared somewhat resistant to lower doses of asparaginase (0.3 U/ml and 1 U/ml respectively). In contrast, healthy control cells (MAPC; Fig. 5F) appeared to recover readily following culture with either arginase or asparaginase. These data suggest that arginase is a more effective cytotoxic agent than asparaginase towards canine lymphoid cell lines.

## Discussion

As previously reported by others [4], the effect of prolonged arginine-deprivation on cells within a cultured malignant cell population is not uniform. Some cells appear obviously dead, some become pyknotic, and some appear to die with their cell membranes intact, able to prevent penetration by trypan blue but otherwise dull and inert. Currently the literature is divided as to the mechanism that induces cell death following arginine deprivation, with convincing evidence to suggest a role for both apoptosis [3] and autophagy [27]. Our own attempts to determine a role for apoptosis using an assay to measure the activity of caspase 3/7 proved unsuccessful because arginase interfered with the assay (data not shown). However, given the heterogeneity in cell death phenotypes, and the lack of adequate cell cycle control in malignant cells, we speculate that the mechanism of cell death in any particular malignant cell in asynchronous cultures is determined by its point in the cell cycle.

The capacity of apparently dead cells to exclude trypan blue, coupled with the fact that we wished to screen adherent osteosarcoma cells over multiple time-points, prompted us to look for an alternative, accurate, and high-throughput measure of the loss of cell viability. We found the quantitation of ATP, an indicator of the energy charge of cells, to be an excellent alternative to trypan blue exclusion, with the added benefit of not requiring trypsinization.

An early report suggested that arginine-deprivation alone was enough to kill >90% of the (predominantly) human malignant cell types tested in vitro (including all of the osteosarcoma- and leukaemia cell lines studied) over the course of 5 days [4]. However, this study did not investigate the possibility that a small number of cells may, in fact, survive and allow the recovery of these malignant cultures given both arginine replenishment and time. Our results suggest, in canine cells at least, that 6 days of arginine-deprivation alone has a less devastating effect on osteosarcomas and lymphoid cells than might be predicted, with 8 out of the 12 lines studied being able to recover following arginine restoration. In agreement with previous studies, however, we found variability in the capacity of our lines to utilize citrulline to maintain cell growth in the absence of supplemental arginine; however, no cells were able to utilize ornithine in this manner [27], [28], [29]. These results suggested that few cells would be able to replace arginine if it could be efficiently depleted, rather than simply excluded from the culture environment.

Our subsequent studies, in which arginase was also added to our cultures to catabolize trace amounts of arginine undetectable using the MassTrak AAA chromatographic method, confirmed arginine depletion to be a more effective and efficient approach to prevent the recovery of these malignant cells. Of the 8 cell lines capable of recovery following 6 days of arginine-deprivation, none were able to recover following arginine-depletion with 10 U/ml of arginase. Indeed, 5 of these 8 lines, including all of the last remaining lymphoid lines, could be rendered non-recoverable following just 3 days of arginase treatment. Interestingly, however, the effects of arginase on the various osteosarcoma cell lines were variable, requiring between 3 and 8 days of treatment to prevent recovery, suggesting that more work is required to identify which osteosarcomas are likely to be susceptible to short-term arginase treatment. Healthy MAPC cells, in contrast, recovered fully following 9 days of arginase treatment. This was expected since, like non-malignant cells deprived of arginine, arginase treatment has been shown to arrest healthy cells in G_1_, a resting stage of the cell cycle from which cells can exit when stimulated [30].

Our observation that arginase appears to have a faster and more devastating effect than asparaginase on canine lymphoid cells in vitro agrees with similar findings reported using mouse L1210 cells [26]. Asparaginase treatment protocols are used for remission induction and intensification treatment in acute lymphoblastic leukemia in all pediatric regimens and in the majority of adult treatment protocols. As a consequence of the requirement for prolonged therapy, often lasting many months, some patients develop neutralizing antibodies to asparaginase, which is often derived from bacteria such as *E. coli*, thereby dramatically reducing the effectiveness of this treatment [31]. Asparaginase administration can also lead to hyperammonemia, pancreatitis, thrombosis, central nervous system complications, liver dysfunction, coagulopathy and anaphylaxis [32], [33], [34]. Arginase may therefore offer an alternative, safer therapeutic enzyme strategy for leukemia patients, particularly as it appears to be effective over a very short time-frame. Indeed, arginase delivery in combination with chemotherapy has recently shown promise in preclinical trials for the treatment of acute lymphoblastic T cell leukemia [35].

This study demonstrates that arginase induces the death of malignant canine cells and prevents their recovery, while at the same time leaving healthy cells unharmed. The utilization of arginase in the treatment of canine lymphomas and osteosarcomas thus warrants further clinical investigation.

## Supporting Information

Figure S1 **Amino Acid analysis of GL-1 supernatant using the MassTrak AAA chromatographic method.** GL-1 cells were cultured for 24 hours as indicated. The amino acid content of culture supernatant was then analysed using the MassTrak AAA chromatographic method. (A) AF alone, or AF media supplemented with 1 mM arginine, 1 mM citrulline or 1 mM ornithine respectively. Error bars represent SD, statistical comparisons relate to comparisons with AF media alone. (B) AF or RPMI media supplemented with 10 U/ml Arginase. Graph shows combined data from three (complete) independent experiments with similar results. Error bars represent SD. ND = Not Detected.
(TIF)

Figure S2 **Sensitivity of canine lymphoid cells and osteosarcomas to short-term arginine-deprivation.** Canine lymphoid cells (CL-1 and CLBL-1) and osteosarcomas (OSCA-71 and OSCA-29) were cultured in Arginine-free media for 3 days with (Blue squares = +1 mM Arginine) or without (Red diamonds = Arginine-free) supplemental arginine. Arginine was reintroduced into cultures on day 3 as indicated by the arrows. Cell viability was determined by ATP quantification. Graphs show a representative experiment from two independent experiments with similar results. Error bars represent SD.
(TIF)
